# Red Display for Three-Color Electrophoretic Displays with High Saturation via a Separation Stage between Black and Red Particles

**DOI:** 10.3390/ma15072555

**Published:** 2022-03-31

**Authors:** Linwei Liu, Wenjun Zeng, Zhengxing Long, Zichuan Yi, Pengfei Bai, Biao Tang, Dong Yuan, Guofu Zhou

**Affiliations:** 1 Guangdong Provincial Key Laboratory of Optical Information Materials and Technology & Institute of Electronic Paper Displays, South China Academy of Advanced Optoelectronics, South China Normal University, Guangzhou 510006, China; yoeksome@sina.com (L.L.); zwjcareer@163.com (W.Z.); mikaellzx@163.com (Z.L.); baipf@scnu.edu.cn (P.B.); b.tang@m.scnu.edu.cn (B.T.); guofu.zhou@m.scnu.edu.cn (G.Z.); 2 College of Electron and Information, University of Electronic Science and Technology of China, Zhongshan Institute, Zongshan 528402, China; 3 Shenzhen Guohua Optoelectronics Tech. Co., Ltd., Shenzhen 518110, China

**Keywords:** electrophoretic displays (EPDs), driving process, red saturation, reference gray scale, electrophoretic particles

## Abstract

A three-color electrophoretic display (EPD) can solve the defect of an insufficient color display of black/white EPDs, but it is difficult to achieve a high red saturation due to the same driving polarity between black and red electrophoretic particles. In this work, a separation stage was proposed in the driving process to increase the red saturation in three-color EPDs. Firstly, red particles’ motion was analyzed by the electrophoretic theory and Stokes’ theorem to optimize driving parameters. Secondly, the activity of black particles was analyzed by testing different driving process parameters, and an optimal activation parameter for red particles was obtained. Next, the separation stage parameters were analyzed to reduce the mixing degree of black and red electrophoretic particles. Experimental results showed that the red and black electrophoretic particles could be effectively separated. Compared with an existing driving method, the red saturation was increased by 23.4%.

## 1. Introduction

As a new type of reflective display, EPD has the advantages of a paper-like reading experience, repeatable eras ability, and bistable characteristics [1,2,3]. There are only two kinds of electrophoretic particles in black/white EPDs, including black particles and white particles. Thus, black/white EPDs have limitations in multi-color image displays [4,5]. In order to realize multi-color displays, three-color EPDs based on new added electrophoretic particles have been proposed [6,7], which enriched the color display of images and meet the demand of the commercial market for multi-color electronic papers [8]. However, red particles are hard to drive due to their large volume, which decreases the red saturation of red images [9].

Red saturation is related to particle movement in microcapsules, which are the basic pixel structure of EPDs, and a good driving scheme design is the key point to increase its value. Driving schemes are composed of different voltage sequences, which are used to display different color states in a pixel [10,11]. Traditional driving schemes of three-color EPDs generally include an erasing stage, an activation stage, and a driving stage [12]. The erasing stage and the activation stage are similar with that of black/white EPDs. The erasing stage is used to erase original images, and ghost images may occur due to its inadequate erasure [13,14]. Thus, an automatic driving scheme generation system was used to recognize ghost images [15], and it could achieve a high-fidelity grayscale. Moreover, the voltage amplitude and driving time of driving schemes obeyed a direct current (DC) balance rule [16,17]. However, the response speed of EPDs could be prolonged when a red driving stage was added. A new DC balance rule was proposed to solve this problem [18], and it could improve the response speed of red particles. The activation stage was used to improve the particle activity and the red saturation [19,20]. However, the red particle could not be activated fully by a traditional activation stage. A new activation stage based on a damping oscillation was proposed [21], and a red particle purification stage was added to separate other particles, but this method did not obey the DC balance rule. A driving scheme based on activation stage optimization was proposed to analyze the effect of driving time on particle activity [22], and different driving time ratio values were tested to form a uniform reference grayscale. A grayscale conversion path algorithm was proposed to reduce driving distance [23], which provided a reference value for increasing red saturation. In addition, the performance of EPDs could also be improved by new composite materials [24,25], a blue nanocomposite coated with an ionic liquid polymer was proposed to promote particle motion [26], and the influence of dispersion medium on particles motion has also been studied [27,28].

In order to increase the red saturation, a new driving scheme was designed by us. First, Stokes’ theorem and the electrophoresis theory were used to analyze the motion of red particles. Then, the effect of particle mixing was characterized by the International Commission on Illumination (CIE) Yxy color space. Finally, a separation stage was designed for increasing the red saturation of three-color EPDs.

## 2. Materials and Methods

### 2.1. Materials of Three-Color EPDs

**Figure 1:**
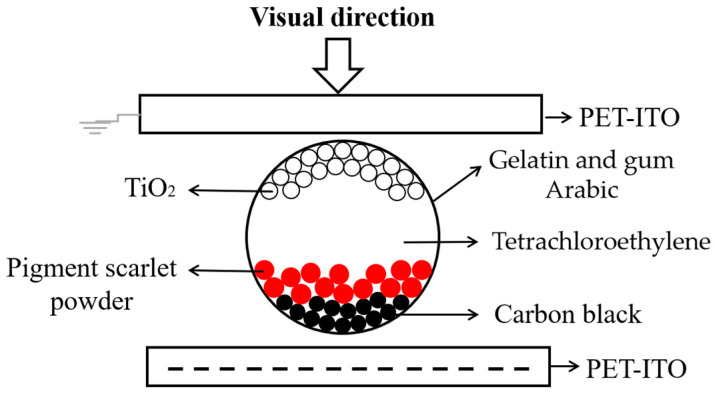
A pixel structure of a three-color EPD. The common electrode is connected to ground, and a negative voltage is applied to the pixel electrode. At this time, white particles are driven to the top of the microcapsule.

Electronic ink (E-ink) is the key material for the display of three-color EPD. E-ink includes electrophoretic particles, a dispersion medium, charge control agent, and stabilizer. There are three kinds of electrophoretic particles in E-ink, which are black, white, and red particles. Specifically, TiO_2_ is used as white electrophoretic particles because of their high reflectivity and excellent optical properties. Carbon black has a strong staining intensity, so it can be used as black particles. Pigment scarlet powder is used as red particles; it is prepared by the diazotization of aniline and coupling with naphthol AS. The dispersion medium is non-polar solvent, and tetrachloroethylene can be used as a medium for the movement of electrophoretic particles. Polyvinyl pyrrolidone can be used as a charge control agent to charge the surface of electrophoretic particles. Span 80 can be used as stabilizer, which is used to prevent the electrophoretic particles from aggregation and sedimentation. The E-ink is separated and wrapped into microcapsules by the complex coacervation method to reduce the interaction force and collision probability between electrophoretic particles. Gelatin and gum Arabic can be used as a microcapsule wall with optical transparency. Polyethylene terephthalate-indium tin oxide (PET-ITO) can be used as the electrode plate of EPD, because PET has good folding properties [29]. The electrode plate is divided into a common electrode and pixel electrode plate, and the common electrode plate is optically transparent. A pixel structure of a three-color EPD is shown in Figure 1 [30,31,32]. The polarities of red and black particles are positive, and the polarity of white particles is negative. When a negative driving voltage is applied, white particles can be driven to the top of microcapsules [33], red and black particles are driven to the bottom of microcapsules, and red particles are above black particles because of their low speed.

### 2.2. Design of Driving Schemes

The driving scheme is used to drive particles, which means it can affect the display quality of EPDs. In a driving process, particles in EPDs are affected by a combination of viscosity and electrostatic forces [34]. The relationship between the electrostatic force and the viscosity can be obtained by the electrophoretic theory and Stokes’ theorem [35]; it is shown in Equation (1).

(1) $$\frac{Uq}{l}-6\pi \eta \nu R=m\frac{d\nu }{dt}\;$$

where U is an applied driving voltage, q is the charge quantity, l is the distance between the common and pixel electrode plates, η is the viscosity coefficient, m is particle mass, and ν is the particle velocity. The first term is electrostatic force, and the second is viscosity. Particles can be driven when the electrostatic force is greater than the viscosity. Their movement is diverse, because their charge, mass, and volume are different. The mass and volume of red particles are the largest among the three types of particles. Driving red particles toward the top of microcapsules is a key point to obtain a high red saturation, and they should not mix with other particles during movement. However, black particles are easily mixed with red particles due to their same charge polarity. In an activation stage, the velocity of particles can be obtained by Equation (2) [36].

(2) $$\nu =\frac{Uq}{6\pi l\eta R}\left(1-e^{-\frac{6\pi \eta R}{m}t}\right)$$

A square wave is generally used as an activation stage in the traditional driving waveform [6], and its voltage amplitude is 30 V. In this stage, black and white particles move faster than red particles, and red particles remain almost stationary. Thus, the activation stage can only separate black and white particles. In fact, red particles can be driven and black particles kept static when a low positive voltage is applied. The reason is that they have a different charge amount. There is a driving voltage, so that the electrostatic force on the red particles is greater than the viscous force, while the electrostatic force on the black particle is not enough. Thus, a new driving scheme could be designed to separate red and black particles based on this feature.

**Figure 2:**
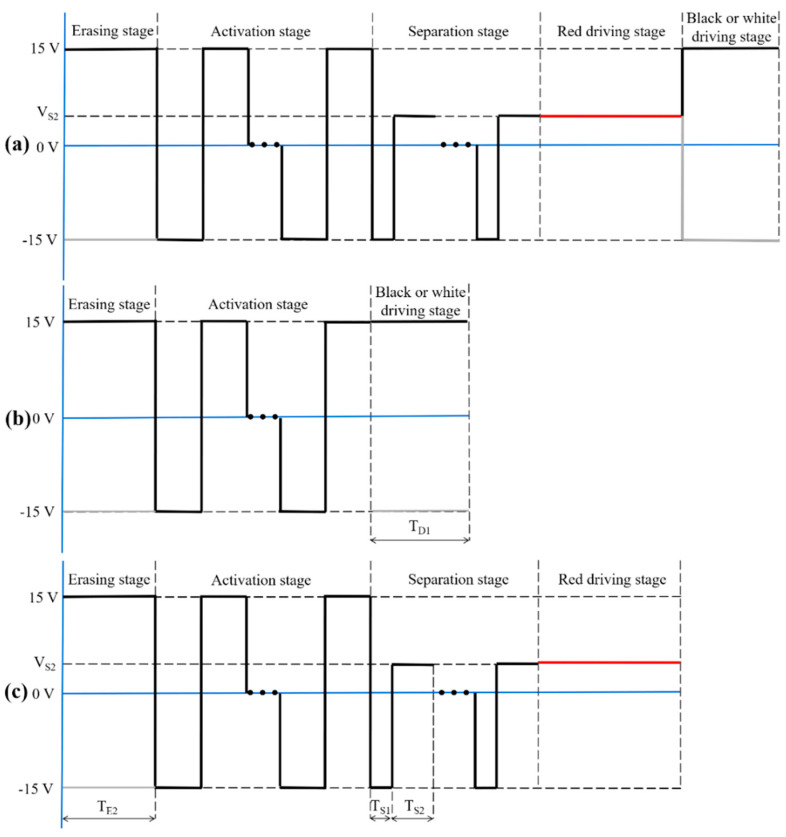
Driving scheme for increasing red saturation. (**a**) A complete structure of a driving scheme. It was composed of an erasing stage, an activation stage, a separation stage, a red driving stage, and a black or white driving stage. (**b**) An example of driving schemes which could be used to display a black image. (**c**) An example of driving schemes which could be used to display a red image.

In order to improve the red saturation, we designed a new driving scheme. It was composed of an erasing stage, an activation stage, a separation stage, a red driving stage, and a black or white driving stage, as shown in Figure 2a. The driving scheme obeyed a voltage balance rule between an original driving scheme and a target driving scheme. For example, an original image was a black image, and it was driven by the original driving scheme, as shown in Figure 2b, and the target image was a red image, and it was driven by the target driving scheme, as shown in Figure 2c. Then, the voltage balance rule was realized between the driving stage of the original driving scheme and the erasing stage of the target driving scheme, which could be expressed by Equation (3).

(3) $$V_{D1}\times T_{D1}+V_{E2}\times T_{E2}=0$$

where $V_{D1}$ and $T_{D1}$ were the driving voltage and driving time of the black or white driving stage in the original driving scheme, respectively; $V_{E2}$ and $T_{E2}$ were the driving voltage and driving time of the erasing stage in the target driving scheme, respectively. The function of the voltage balance rule was to avoid screen damage caused by charge residue. In the activation stage, an adjustable square wave was used to separate black and white particles, and a black reference grayscale could be formed at the end of this stage. The black reference could decrease the moving distance of red particles for displaying a red image. Then, a separation stage was designed to separate black and red particles. Black particles were driven toward the lower part of microcapsules when the voltage was −15 V; then, red particles could be driven toward the upper part of microcapsules when the voltage was $V_{S2}$. The driving time of the two voltages could be calculated by Equation (4).

(4) $$-15\times T_{S1}+V_{S2}\times T_{S2}=0$$

where $T_{S1}$ was the driving time of −15 V applied in the separation stage, and $T_{S2}$ was the driving time of $V_{S2}$. The distance between red particles and black particles was increased when several cycles of −15 V and $V_{S2}$ were applied. Thus, black and red particles could be separated at the end of this stage. Finally, a red driving stage was applied to display a red image; the driving voltage of this stage was equal to $V_{S2}$.

### 2.3. Experimental Method

The experiment was conducted to test the performance of driving schemes, including the red saturation and luminance curves of the three-color EPD. Firstly, voltage sequences designed by driving schemes were edited by the Arbexpress waveform editing software (V3.4, Tektronix, Beaverton, OR, USA), and the voltage amplitude was set to 3 V. Then, the edited voltage sequence was imported into a function generator (AFG3022C, Tektronix, Beaverton, OR, USA); the high limit and the low limit voltage of the function generator were 5 V and −5 V, respectively. Next, the output of the function generator was connected with the input of a voltage amplifier (ATA-2022H, Agitek, Xi’an, China). The voltage gain of the voltage amplifier was set to 10, and the input resistance and output resistance were set to 50 Ω and 5 Ω, respectively. Moreover, the voltage amplifier was connected with a three-color EPD (Dalian Longning Technology Co., Ltd., Dalian, China); parameters of the three-color EPD are shown in Table 1. Further, a colorimeter (Arges-45, Admesy, Ittervoort, The Netherlands) was placed on the three-color EPD. The tristimulus of the EPD could be measured by the colorimeter in the spectral range of 380–780 nm, and then, tristimulus values were converted into CIE Yxy color space values. The red saturation of the three-color EPD could be characterized by an x value of CIE Yxy, and the luminance could be characterized by a Y value of CIE Yxy. Thus, the red saturation and the luminance values described in this paper were an x value and Y value of CIE Yxy, respectively. Finally, the tested data were transmitted to a computer (H430, Lenovo, Beijing, China) by a universal serial bus (USB) interface, and data were recorded to analyze the performance of driving schemes.

## 3. Results and Discussion

### 3.1. Driving Scheme Parameters Analysis

**Figure 3:**
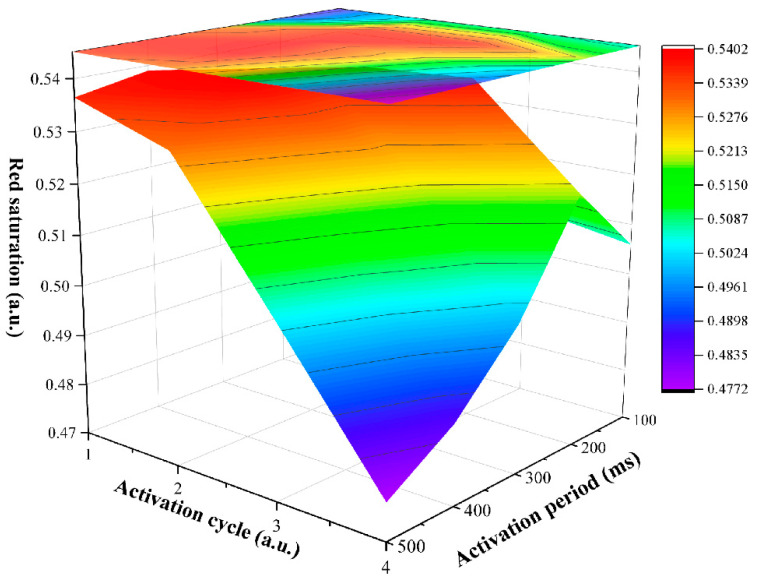
The relationship between red saturation and activation stage parameters. When the activation cycle was a constant, the red saturation was increased first and then decreased with the increase of the activation period. The red saturation had the same trend with the increase of the activation cycle. The maximum value was 0.54 when the activation period was 300 ms and the activation cycle was 2. The minimum value was 0.47 when the activation period was 500 ms and the activation cycle was 4.

The effect of different activation stage parameters on red saturation were studied. An original image and a target image were set to black and red, respectively. The driving time was set to 200 ms, and the driving voltage was set to −15 V in the erasing stage. The period and cycle of the separation stage were set to 60 ms and 30, respectively. The driving time was set to 3 s, and the driving voltage was set to 3 V in the red driving stage. In the activation stage, the period was set to 100, 200, 300, 400, and 500 ms, and then, the cycle was set to 1, 2, 3, and 4. Plots of red saturation driven by different activation stage parameters are shown in Figure 3. There was a maximum value of red saturation when the activation period or cycle was a constant, and this maximum value generally did not appear at a starting point or an end point. Specifically, the red saturation was increased first and then decreased with the increase in the activation period when the activation cycle was a constant. Moreover, the position of the maximum value tended to the direction of less of an activation cycle. This trend was consistent when the activation cycle was a constant. This indicated that the activity of black particles was increased when the activation period and cycle were increased, and it could not be separated by the separation stage. Then, a low red saturation in the red driving stage was caused because black particles and red particles were mixed. Overall, the red saturation showed a slow downward trend with the increase of the activation period and cycle. The red saturation reached the maximum value when the activation period was 300 ms and the activation cycle was 2. At this point, the activation stage could form an optimal reference black grayscale for driving red particles.

**Figure 4:**
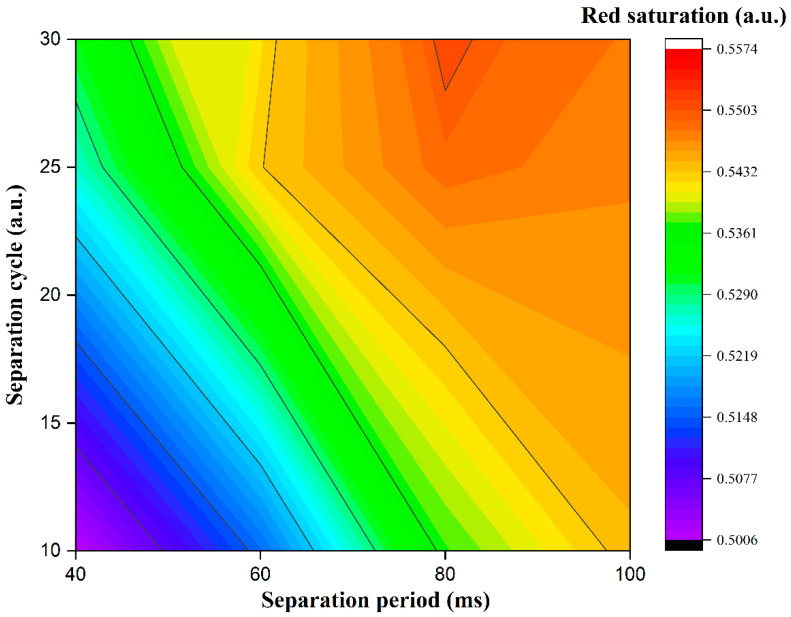
The relationship between red saturation values and separation stage parameters. The red saturation had an increasing trend when the separation period and cycle were increased. The maximum value was 0.55 when the separation period was 80 ms and the separation cycle was 30. The minimum value was 0.50 when the separation period was 40 ms and the separation cycle was 10.

Separation stage parameters were studied to achieve a high red saturation. In the activation stage, the period was set to 300 ms, and the cycle was set to 2. The separation period of the separation stage was set to 40, 60, 80, and 100 ms, and the separation cycle was set to 10, 20, and 30. Plots of red saturation values driven by different separation stage parameters are shown in Figure 4. The red saturation had an increasing trend when the separation period and cycle were increased. Particularly, the red saturation was proportional to the separation period when the separation cycle was 10. This was because the separation between black particles and red particles was insufficient when the separation period was short. However, this trend was changed when the separation cycle was 20 and 30; the red saturation was decreased when the separation period reached 100 ms. The duration of −15 V was increased when the separation period was long. At this time, black particles were driven to the bottom of microcapsules, which made them close to red particles. Thus, the red saturation could be decreased when the separation period was too long. When the separation period was a constant, the red saturation was positively correlated with the separation cycle. This indicated that the distance between red particles and black particles could be increased when the separation stage was applied repeatedly. However, the driving time was proportional to the separation cycle. Therefore, the separation cycle should be set in an appropriate range. The red saturation reached the maximum value when the separation period and cycle were 80 ms and 30, respectively. At this point, red particles and black particles could be separated fully.

### 3.2. Performance Comparison of Driving Schemes

**Figure 5:**
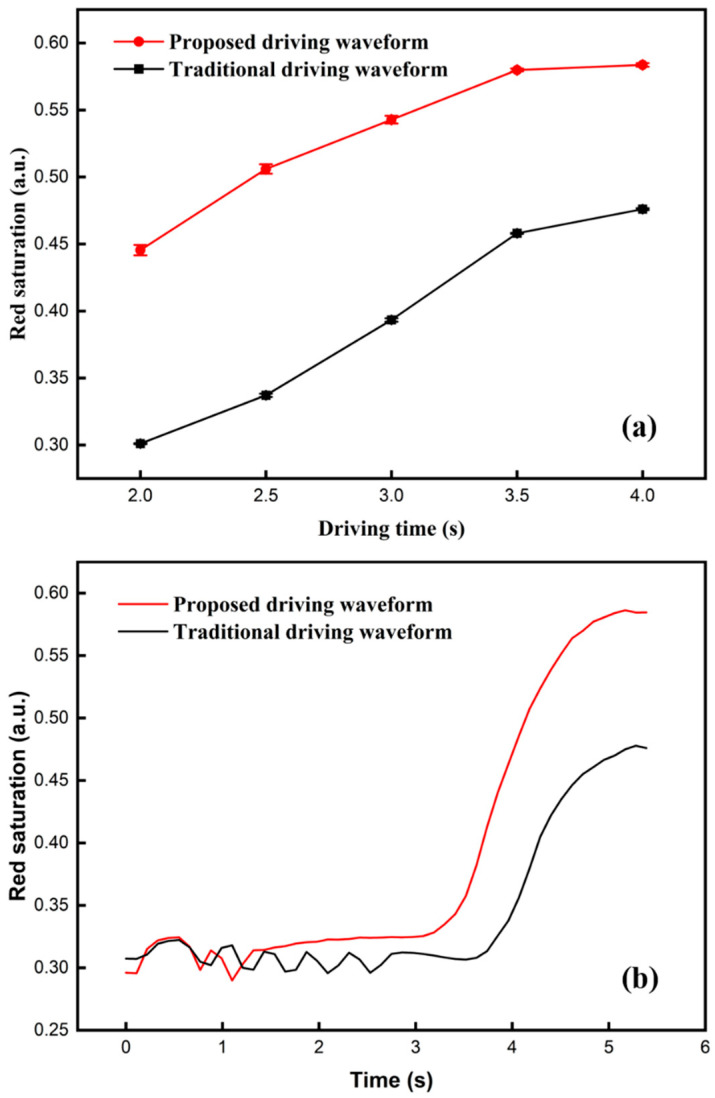
The red saturation of the three-color EPD with different driving schemes. (**a**) The relationship between the red saturation and different driving times of the red driving stage. (**b**) Red saturation curves of the traditional and the proposed driving scheme when the driving time was 4 s. The maximum value of red saturation was 0.58, which was 0.11 higher than that of the traditional driving scheme.

A traditional driving scheme [6] was used for comparison in the experiment. In the activation stage of the traditional driving scheme, the period was set to 400 ms, and the cycle was set to 5; the driving voltage of the red driving stage was 3 V. In the activation stage of our proposed driving scheme, the period was set to 300 ms and the cycle was set to 2. In the separation stage of the proposed driving scheme, the period was set to 80 ms, and the cycle was set to 30. The driving time of the red driving stage were both set to 2, 2.5, 3, 3.5, and 4 s. The relationship between the red saturation and driving time is shown in Figure 5a. The results showed that the red saturation of the traditional driving scheme (black line) and the proposed driving scheme (red line) were both positively correlated to the driving time. In the same driving time, the red line was higher than that of the black line. The maximum and minimum value of the proposed driving scheme were 0.58 and 0.44, respectively. On the contrary, the maximum and minimum value of the traditional driving scheme were 0.47 and 0.30, respectively. In the traditional driving scheme, the red particles could be driven to the bottom of microcapsules due to its DC balance rule design when the erasing stage was applied, and red and black particles could not be separated by its activation stage.

**Figure 6:**
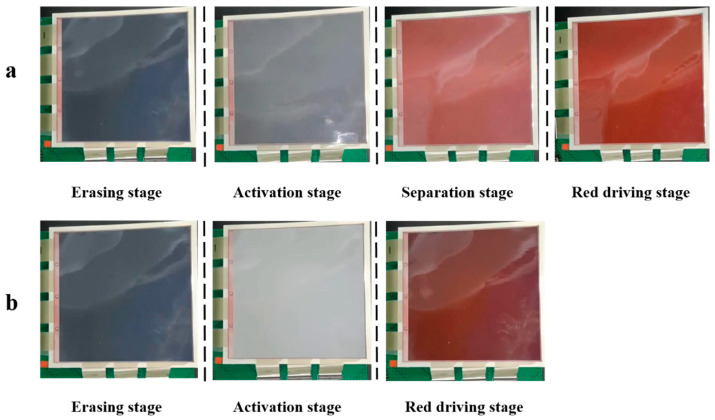
Images of the three-color EPD in each stage when it was driven by the two driving schemes. (**a**) It was driven by the proposed driving scheme. (**b**) It was driven by the traditional driving scheme.

In particular, curves of the red saturation changed with time and are shown in Figure 5b. Black and red lines represented red saturation curves of the traditional driving scheme and the proposed driving scheme, respectively. The red saturation of the black line was maintained in a low range when the time was in the range of 0–3.5 s. The distance between red particles and the top of microcapsules was increased when a reference grayscale was formed. Then, the red saturation was increased slowly due to the long distance and the mixing of particles. The red saturation of the red line was maintained in a low range when the time was in the range of 0–3 s. The response time could be reduced because the erasing stage was shortened by the optimization of balance rule. The difference in the driving time between the two curves was about 0.99 s when the red saturation was 0.47. In addition, it is worth noting that the red saturation of the red line had an upward trend when the time was in the range of 1.3–3 s. This indicated that red particles had a tendency to move upwards in microcapsules, and black particles and red particles could be separated by the separation stage. Then, the red saturation could be increased when the red driving stage was applied, the increasing rate of the red line was faster than that of the black line. Finally, experimental results showed that the red saturation could be increased by 23.4% compared with the traditional driving scheme. Images of three-color EPDs in each stage are shown in Figure 6. The image of the proposed driving scheme could display red color in the separation stage, and the final image showed a higher purity red color compared to the final image formed by the traditional driving scheme. Therefore, the driving scheme proposed by us could effectively increase the red saturation.

## 4. Conclusions

In this paper, a driving method was proposed for increasing red saturation in three-color EPDs. First, an optimal black reference grayscale was formed by optimizing the activation period and cycle in the activation stage. Then, red particles and black particles could be separated adequately by the separation stage, and an optimal separation period and cycle were determined. Finally, the experimental results showed that the red saturation was increased and that the response time was decreased compared with a traditional driving scheme. In summary, we proposed a driving method design for solving the problem of low red saturation in three-color EPDs, which provides valuable design ideas for the driving theory and system design of electronic displays.

## Figures and Tables

**Table 1:** Parameters of the three-color EPD.

$\mathrm{Panel}\;\mathrm{Size}\;({\mathrm{cm}}^{2}$ **)**	**Color State**	Resolution	$\mathrm{Pixel}\;\mathrm{Size}\;({\mathrm{cm}}^{2})$	Voltage (V)	Response Time (s)
11.5×11.5	Black, white, red	20×20	0.5×0.5	≤20	≤5

## Data Availability

All data contained within the article.
